# Report upon a Case of Death from Chloroform

**Published:** 1853-11

**Authors:** 


### SELECTIONS.

From the London Lancet

### *Report upon a Case of Death from Chloroform.*

[Communicated to the Society of Surgery, by M. DE VALLET, Surgeon-in-Chief to the Hotel-Dieu, d'Orleans.]

A soldier of the line, aged 25, apparently in good health, and of strong frame, consulted M. Vallet for a small tumour (encysted?) situated behind the right labial commissure. Before operating, M. Vallet proceeded to direct the inhalation of chloroform. The patient fasting, being placed in the horizontal posture, the chloroform (about one gramme, or gr. xx.) was poured upon a hollow sponge and applied to the nose, the mouth being left free. At the expiration of a minute, no effect having been produced, four grammes (rather more than a drachm and a quarter) were poured on the sponge, and, at the expiration of four minutes, the patient, without having experienced any irritation of the larynx, without having manifested any resistance, without redness of the countenance, and after only a slight period of agitation, fell into a state of insensibility fit for the operation. Scarcely had the incision been made necessary to expose the cyst, when the patient became pale, respiration was suspended, and he sank into a state of extreme collapse. All the usuall remedies were tried, and without avail. M. Vallet opened the trachea, and performed artificial respiration with an elastic tube; then an electric current was sent by needles through the region of the heart. The patient died without any sign of reaction.

*Examination of the body.*—The vessels of the brain were empty; the lungs were congested with blood, which in some situations was extravasated; the heart was excessively flaccid; there were some soft clots in the right cavities; the left were empty. The stomach was full of gas; the liver, spleen, and kidneys were gorged with black blood. The blood from the subclavian veins was analysed by an experienced chemist, but no trace of chloroform was detected.

Death, according to the author, seems to ensue under two conditions:—

1. It is preceded by symptoms resembling those of asphyxia, when first respiration, and then circulation cease.

2. Life seems extinguished yet more quickly in profound syncope.

A case of death, occurring under the latter circumstance, was mentioned by M. Gorre, of Boulogne, and communicated to the Academy, 1848. A lady, aged 30, requiring a fistulous passage to be laid open, inhaled chloroform from a handkerchief, upon which about twenty drops had been poured. Scarcely had she inspired. when she exclaimed, "I am suffocated." The face became pale, the features changed, respiration was embarrassed, and froth came to the mouth. Less than a minute after the first administration of the chloroform, the handkerchief was withdrawn, and the surgeon proceeded with the operation, believing the symptoms were transitory, but life was soon found to be extinct.

In this case, as in the one already related, there were found, upon *post-mortem* examination, congestion of the lungs, and excessive flaccidity of the heart. The cerebral vessels were empty.

M. Jobert (de Lamballe) has read before the Society a well drawn up *Memoire* upon the subject of Anæsthetics. We think him rather unfair to our distinguished countryman, Dr. Simpson, to whom the merit of introducing chloroform in surgery most undoubtedly belongs, when he remarks: — "M. Fleurene in France, and M. Simpson in England, have introduced into science a most valuable anæsthetic, chloroform; the first by his experiments upon animals; the second by his administration of it to man." We cannot allow this "sharing of the honor;" and we are convinced M. Jobert will acknowledge it to be rather disingenuous. The greater part of his conclusions are not new; they are those generally adopted in this country. But, in comparing chloroform and ether, he remarks: —

"Ether irritates the passages which it traverses, is disagreeable to the patient, and excites cough. Chloroform produces no irritation, and is pleasant (?) to those who use it. Chloroform produces but feeble organic muscular irritation; ether excites it violently, since its inspiration causes agitation of the heart and other muscles. Ether provokes its anæsthetic effects slowly, and they are prolonged over some time, under the form of intoxication, pain in the head, small pulse, and coldness of the body. Chloroform, however, ceases acting after the removal of the apparatus. Ether changes the color and consistence of the blood. Such is not the case with chloroform. Chloroform does not interfere with the cicatrization of the wound; ether renders it slow, by diminishing the consistence of the plastic lymph.

"Ether excites the organs of generation; chloroform does not. Ether can, with difficulty, produce death; such is not the case with chloroform, which can make life cease instantaneously when the patient is not very closely watched. He infers that ether is preferable to chloroform, in cases where there is great depression of the nervous system from sudden injury, as after a gun-shot wound; in cases where there has been a long and abundant suppuration, great loss of blood, or where the chlorotic condition is in an advanced state."

We understand that Mr. Stanley, of St. Bartholomew's Hospital, is now giving sulphuric ether a more extended trial, in producing insensibility upon patients about to undergo operations; and perhaps it may be found that there are cases specially adapted to both preparations. Chloric ether, too, — at one time used as an anæsthetic extensively by Mr. Lawrence in private practice — should not be forgotten; it can be inhaled readily in cases where sulphuric ether excites the most uncontrollable cough.

